# The characteristics of peripheral lung tumours that suggest their bronchiolo-alveolar origin.

**DOI:** 10.1038/bjc.1968.77

**Published:** 1968-12

**Authors:** O. Campobasso

## Abstract

**Images:**


### THE CHARACTERISTICS OF PERIPHERAL LUNG TUMOURS THAT SUGGEST THEIR BRONCHIOLO-ALVEOLAR ORIGIN

O. CAMPOBASSO

*From the Institute of Morbid Anatomy and Histology, University of Turin, Turin, Italy*

Received for publication July 12, 1968.

TUMOURS of the lower respiratory tract have generally been regarded as bronchial tumours. However, some authors (Garland, Beier, Coulson, Heald and Stein, 1962; Lisa, Trinidad and Rosenblatt, 1965) have pointed out recently that many pulmonary tumours have a peripheral origin. A similar conclusion was reached by Mottura and Campobasso (1966) who described the histological features of 557 tumours of the lung, studied on postmortem specimens and surgical resections. They concluded that these tumours could be classified histologically under four headings: anaplastic small cell carcinoma; squamous cell carcinoma; anaplastic large cell carcinoma and adenocarcinoma. This classification is not new and is very similar to that used by other workers (Fischer, 1949; McDonald, McBurney, Carlisle and Patton, 1951; Walter and Pryce, 1955; Whitwell, 1961; Delarue, Abelanet and Paillas, 1964), but the recent work has provided a new histogenetic basis for the classification, which suggests that among the tumours of the lower respiratory tract, a distinction should be made between the true bronchogenic carcinomas, which include anaplastic small cell carcinoma and squamous cell carcinoma, and the bronchiolo-alveolar carcinomas which include anaplastic large cell carcinoma and adenocarcinoma, the latter being tumours of the lung parenchyma.

On the other hand, bronchial epithelium is, in the main, a lining epithelium and its histological and ultrastructural appearances are different from bronchiolar and alveolar epithelium (Krahl, 1963; Collet, 1965). The latter are directly concerned with respiratory function and are responsible for the growth and regeneration of pulmonary tissue, both in foetal and post-natal life (Willson, 1928; Amprino, 1937; Dunnill, 1962). Consequently, it seems logical to assume that if two kinds of epithelium exist, two kinds of tumour also exist, each related to the respective type of epithelium of origin.

The purpose of this present paper is to illustrate the significance of the relationship between the location of the tumour and its histological type, and to describe and to discuss some histological aspects of peripheral lung tumours. This has provided more evidence to show that tumours of the lower respiratory tract may well be of two histogenetic patterns.

### MATERIALS AND METHODS

Five hundred and thirty-one tumours were obtained by surgical resection at the Thoracic Surgery Centre of the University of Turin. The tumours were sub-divided into hilar-parahilar and peripheral tumours, according to whether or not they were in obvious connection with the bronchial tree. To establish whether or not this connection existed, all the bronchial branches were cut and opened with thin scissors as far peripherally as possible. It was easy to recognise the tumours connected with the bronchi, but it was more difficult to exclude those which were not. The tumours which were found to be connected with the bronchial branches, smaller than 2 mm. in diameter were regarded as being peripheral. In many tumours it was impossible to establish the exact point of origin; they were connected with intra-segmental bronchial branches, but it was very difficult to say whether the connection was the result of their origin at the site or due to secondary neoplastic invasion. Such tumours have been classified as " uncertain ".

It was found that the relationship between the location and the histological pattern of the tumour was able to be studied in detail in 414 (78 per cent) of the specimens. The cases where details of either macroscopic or microscopic appearances were not available or where the specimen was either an obvious or possible metastasis were excluded from the present investigation. In addition, 5 tumours of mixed squamous and adenomatous carcinoma and 2 tumours which had probably originated in the bronchial glands were excluded from this present investigation.

The specimens were fixed in 10 per cent formalin; 4 to 5 blocks were prepared from different parts of each specimen and embedded in paraffin; the sections were cut at approximately 7 microns and stained with haematoxylin and eosin. According to the criteria used by Mottura and Campobasso (1966), tumours were classified under four histological headings, as follows: anaplastic small cell carcinoma; squamous cell carcinoma; anaplastic large cell carcinoma, and adenocarcinoma.

### RESULTS

The relationship between the location of pulmonary tumours in respect to the bronchi and their histological types are summarised in Table I. It can be seen

TABLE 1.—*Relationship Between the Location of Lung Tumours and Their Histological Type*

| Histological type | Total number | Hilar or parahilar (in connection with the bronchi) | | Uncertain | | Peripheral (without any connection with the bronchi) | |
|---|---|---|---|---|---|---|---|
| | | No. | per cent | No. | per cent | No. | per cent |
| Small cell carcinoma | 45 | 36 | (80·0) | 8 | (17·8) | 1 | (2·20) |
| Squamous cell carcinoma | 222 | 161 | (72·5) | 44 | (19·8) | 17 | (7·7) |
| Large cell carcinoma | 87 | 6 | (6·9) | 20 | (22·9) | 61 | (70·2) |
| Adenocarcinoma | 60 | 3 | (5·0) | 12 | (20·0) | 45 | (75·0) |

that there are many anaplastic small cell carcinomas and squamous cell carcinomas among the hilar and parahilar tumours and many anaplastic large cell carcinomas and adenocarcinomas that are situated peripherally. On the other hand, only one anaplastic small cell carcinoma and few (7·7 per cent) squamous cell carcinomas were sited peripherally and a few anaplastic large cell carcinomas (6·9 per cent) and adenocarcinomas (5 per cent) were observed to be in intimate connection with the bronchi. It was found that the precise point of origin of about 20 per cent of the tumours of each of the four main histological types could not be determined with any accuracy; these are referred to as " uncertain " in Table I.

Examination of the microscopical appearance of the tumours gave important indications which were helpful in trying to determine the histogenesis of the peripheral tumours. In the central areas of many of the adenocarcinomata it was possible to see, in the glandular spaces, alveolar macrophages containing granules of haemosiderin or carbon in their cytoplasm (Fig. 1, 2 and 3).

In some peripheral tumours it was possible to see features of organised pneumonia, *i.e.* alveolar-like spaces filled with masses of fibrin and bundles of fibroblasts, joined by bridges passing through Kohn's pores (Fig. 4). This is strongly suggestive that the tumour was of peripheral origin and originally had atypical alveolar spaces within its structure and that these have undergone a pneumonic, inflammatory change and have become organised. The arrangement of the fibroblasts, inflammatory cells and fibrin seen in these lesions was typical of a recent inflammatory change and did not suggest that the neoplasm had invaded an area of pre-existing chronic pneumonitis.

Some of the peripheral tumours resembled foetal lung. There are three main phases during the embryonic and foetal development of the lung (Dubreuil, LaCoste and Raymond, 1936; Loosli and Potter, 1951): firstly, glandular until the 16th week of intra-uterine life, during which time the bronchial branches are formed; canalicular from the 16th to the 24th week, when there is the period of the formation of terminal bronchioli and, finally, alveolar from the 24th week until birth, which is the period for the formation of the respiratory bronchioli and the alveoli.

The tumours which had histological features of the foetal lung were either comparable to the alveolar phase (Fig. 5 and 6) or were of similar structure to the foetal lung in its canalicular phase (Fig. 7 and 8).

An intimate admixture of the various cell types was frequently seen in certain forms of lung tumours. However, anaplastic small and large cells were never seen together in the same tumour, and very seldom was a true squamous cancer of the lung observed in areas of adenocarcinomatous growth. On the contrary, in many instances, areas of large cell carcinoma were seen to be in close association with adenocarcinomatous areas, and small cell and squamous carcinomas were likewise often observed to be intermixed. It is also of interest that many anaplastic large cell carcinomas consisted of large, clear cells which resembled those of adenocarcinomas as well as those which lined alveolar cavities during foetal development (Fig. 9, 10 and 11).

Previous workers (Nash and Stout, 1958; Hellstrom and Fisher, 1963; Friedberg, 1965; Guillan and Zelman, 1966) have found it difficult to establish the exact point of origin of the giant cell carcinomas, most of which are peripheral. In the present series, features suggesting that most large cell and giant cell carcinomas develop from the epithelium of terminal or respiratory bronchioli have been noted (Fig. 12 and 13). This observation has been reported previously in the giant cell carcinoma (Campobasso and Ferrara, 1963).

### DISCUSSION

The figures shown in Table I clearly demonstrate that the histological features of lung tumours are quite different, according to whether or not the tumour is in obvious connection with the bronchi.

The constant, intimate relationship between the anaplastic small cell and squamous cell carcinomas and the bronchi is consistent with the concept that these tumours develop from bronchial pseudostratified, columnar epithelium. This epithelium has a basal layer and can easily undergo squamous metaplasia. It is, therefore, easy to explain the occurrence of anaplastic small cell carcinomas and squamous cell carcinomas as well as the occurrence of intermediate lesions—transitional cell carcinoma, according to Reid and Carr (1961).

On the contrary, the peripheral site of most anaplastic large cell carcinomas and adenocarcinomas strongly suggests that these tumours do not develop from bronchial epithelium.

The presence of macrophages, containing granules of haemosiderin or carbon and more defined inflammatory changes within lung adenocarcinomas, as well as morphological similarities between these tumours and foetal lung, have never been observed in squamous cell or anaplastic small cell carcinomas. These macrophages are characteristic of pulmonary alveolar cavities; since their life-span is from 7–8 days (Spencer and Shorter, 1962), it is hard to imagine that they are pre-existing macrophages which survived in the neoplastic tissue. It is more logical to assume that they have migrated from the connective tissue into these cavities following their formation by the neoplastic growth. These cavities are, therefore, not likely to be related just to neoplastic invasion of pulmonary parenchyma, and their structure supports the concept that the cavities forming lung adenocarcinomas are not glandular cavities but atypical alveolar or bronchiolar spaces. If this interpretation is accepted, it follows that these tumours arise from bronchiolo-alveolar epithelium, since respiratory parenchyma is exclusively formed by this epithelium from the 16th week of intra-uterine life. Moreover,

---

EXPLANATION OF PLATES

FIG. 1.—Peripheral adenocarcinoma of the lung: the glandular spaces contain large dark cells. H. & E. × 210.

FIG. 2.—Higher magnification of the tumour shown in Fig. 1: the large dark cells inside the glandular spaces are macrophages containing granules of carbon dust. H. & E. × 500.

FIG. 3.—Peripheral adenocarcinoma of the lung: the glandular spaces contain dark cells, some of which appear to pass through the wall (arrows.) H. & E. × 210.

FIG. 4.—Inflammatory changes in peripheral adenocarcinoma of the lung: glandular spaces are filled with masses of fibrin joined by bridges passing through Kohn's pores. H. & E. × 100.

FIG. 5.—Foetal human lung at the beginning of the alveolar phase (crown-rump length = 23 cm.): alveolar spaces are lined by clear cuboidal cells. H. & E. × 210.

FIG. 6.—Peripheral adenocarcinoma of the lung: glandular-like cavities are lined by clear cuboidal cells with dark nuclei. H. & E. × 210.

FIG. 7.—Human foetal lung in the canalicular phase (crown-rump length = 14 cm.): many bronchioli with short ramifications are surrounded by an abundant mesenchyma. H. & E. × 100.

FIG. 8.—Peripheral adenocarcinoma of the lung: bronchiolar-like structures with short ramifications are surrounded by an abundant stroma resembling the foetal mesenchyma. H. & E. × 210.

FIG. 9.—Foetal human lung in the alveolar phase. H. & E. × 600.

FIG. 10.—Peripheral adenocarcinoma of the lung. H. & E. × 600.

Fig. 11.—Anaplastic large cell carcinoma of the lung. H. & E. × 600.

FIG. 12.—Anaplastic large cell carcinoma of the lung: the neoplastic cells are in continuity with the epithelium of a bronchiolus. H. & E. × 210.

FIG. 13.—Giant cell carcinoma of the lung: neoplastic growth of the epithelium of a bronchiolus. H. & E. × 210.

(From Campobasso and Ferrara, Cancro, 1963.)

1

2

3

Campobasso.

4

5

6

Campobasso.

7

8

Campobasso.

9

10

11

12

13

Campobasso.

since neither glands nor mucus-secreting cells exist in the periphery of the lung, it would be very difficult to explain the histogenesis of a true peripheral adenocarcinoma of the lung.

The bronchiolo-alveolar cells do not, therefore, only give rise to pulmonary adenomatosis and to that ill-defined entity that goes under the name of bronchiolo-alveolar carcinoma but to all pulmonary tumours of glandular structure.

Some pathologists (Hellstrom and Fisher, 1963; Melnick, Rosen, Howell and Heiser, 1964; Friedberg, 1965; Herman, Bullock and Waken, 1966) using histochemical stains, concluded that many anaplastic large cell carcinomas, as well as giant cell carcinomas, can be classified as adenocarcinomas. Many other features illustrated in this paper, the peripheral site of both types of tumour, the similarity of their cells and the frequent intermingling of the two histological appearances, are suggestive that the anaplastic large cell carcinomas, including the giant cell type, are histologically related to the adenocarcinomas and must, therefore, be considered as undifferentiated carcinomas of bronchiolo-alveolar origin. This origin may sometimes be demonstrated in sections (Fig. 12 and 13). Furthermore, osmiophilic lamellated bodies in the cells of 2 anaplastic large cell carcinomas, as well as in the cells of 1 adenocarcinoma were recently demonstrated by electron microscopy (Mollo, Campobasso and Canese, 1967).

The following classification for pulmonary tumours is, therefore, suggested:

*Bronchogenic carcinomas*

(a) undifferentiated: small cell carcinoma (round and oat-cell carcinoma);
(b) differentiated: squamous cell carcinoma (with or without keratinisation).

*Bronchiolo-alveolar carcinomas*

(a) undifferentiated: large cell carcinoma (including giant cell carcinoma);
(b) differentiated: adenocarcinoma (carcinoma with bronchiolar and/or alveolar features).

It can be speculated that the polymorphic aspects of the peripheral tumours of the lung could be due to the different types of the cells which line the most distal ramifications of the air passages; in addition to the cuboid cells of the bronchioli, an alveolar epithelium exists. Ultrastructural studies of the alveolar epithelium, recently summarised by Brooks (1966), help to distinguish two types of cell (small and large cells of Policard, Collet and Pergermain, 1957) with different morphology and function.

Certain points do not wholly agree with the proposed classification of pulmonary tumours: the existence of some peripheral squamous cell tumours other than an occasional anaplastic small cell carcinoma (Table I); peripheral squamous cell carcinomas are rare when the diagnosis is based on the following criteria: laminae of epithelium, the cells of which show a palisade pattern at the periphery and a distinct differentiation in layers, well-developed horny pearls and intercellular bridges, although the latter have been seen only rarely in the author's experience. Squamous cell carcinomas are usually diagnosed more frequently when cytological evidence is considered alone—that is, on the presence of polygonal cells with large, intensely eosinophilic cytoplasm, which appears keratinised. These tumours should be regarded as large cell anaplastic carcinomas in which regressive changes simulate keratinisation (Mottura and Campobasso, 1966). However, occasionally true squamous cell carcinomas develop at the periphery of the lung.

The bronchiolo-alveolar epithelium has the same embryological origin as that of the bronchial epithelium. Waddell (1949) suggested that they both arise independently from mesoderm. However, other authors (Amprino, 1937; Bucher and Reid, 1961a; Leeson and Leeson, 1964; Brooks, 1966) regard them as of endodermal origin and consider that bronchiolo-alveolar epithelium derives directly from the bronchial epithelium when the distal extremities of the bronchi begin to form bronchioli and the respiratory part of the lung parenchyma. Therefore, even if the bronchiolo-alveolar epithelium becomes especially differentiated from the 4th to the 5th month of intra-uterine life, it is not unreasonable to suppose that it will preserve certain characteristics of tracheo-bronchial epithelium. The possibility that alveolar epithelium, in certain conditions, undergoes squamous metaplasia and subsequently gives rise to squamous carcinoma appears to be supported by study of experimental lung tumours in mice (Kotin and Wiseley, 1963).

The apparently primary localisation of the anaplastic large cell carcinomas and of adenocarcinomas in the large bronchi (Table I) can be easily explained by the secondary invasion of the hilar structures, as has already been suggested both in human (Raeburn and Spencer, 1953) and experimental (Stewart, 1959) lung tumours. The alveolar parenchyma exists even around the segmental and lobar bronchi; a tumour developing in this tissue could rapidly infiltrate the adjacent bronchi, protrude into the lumen and simulate a tumour of bronchial origin. In the present series this has occurred in less than 5–7 per cent of cases (see Table I). Another possible explanation of the occasional presence of carcinomas of glandular structure in the main bronchi is that these are metastases from a primary adenocarcinoma in another site; this has been observed in adenocarcinoma of the rectum. Alternatively, tumours originating in the mucous glands of the bronchial walls which, even though rare, can have the appearance of muco-epidermoid carcinomas (Kreyberg, 1962).

The mucus-secreting character of the cells in certain peripheral carcinomas, either solid or glandular, contrasts with the fact that, under normal conditions, mucus-secreting cells in bronchioli and alveoli cannot be detected by either light or electron-microscopy, in the foetal as well as in the adult lung (Schulz, 1959; Bucher and Reid, 1961b; Krahl, 1963; Leeson and Leeson, 1964). On the other hand, columnar mucus-secreting cells have been observed in cases of pulmonary adenomatosis in which, with serial sections, the alveolar origin of the neoplasm could be ascertained (Rosemond, Boucot and Aegerter, 1951; Campobasso, 1963). The presence of mucus-secreting cells of alveolar origin in pulmonary adenomatosis have been confirmed by Schulz (1963) with histochemical and ultrastructural studies. Cells of this type line the alveolar cavities in the so-called congenital cystic adenomatoid malformation (Belanger, La Fleche and Picard, 1964). All these findings suggest that, under certain conditions, the alveolar and bronchiolar cells can undergo mucus-secreting transformation as well as squamous metaplasia.

The outcome of this investigation has suggested that the following reasoning can be logically applied to determining the histogenesis of lung cancers. There are true cancers of pulmonary parenchyma developing from bronchiolar and alveolar epithelium and they are not uncommon. Although they often have an adenocarcinomatous pattern, they are atypical growths of pulmonary tissue and can be more or less differentiated: they range from the typical pulmonary adenomatosis to the most anaplastic carcinoma—that is the giant cell carcinoma. The relationship between peripheral adenocarcinomas of the lung and bronchiolo-alveolar carcinomas is thus made clear. In fact, in spite of the possible presence of mucous cells in the tumour, since there are no glandular structures in the normal pulmonary parenchyma, a true pulmonary adenocarcinoma—that is a carcinoma which develops from a glandular epithelium—does not exist. Thus, all peripheral lung tumours must be considered as bronchiolo-alveolar tumours, whether they reproduce structures such as bronchioli or alveoli or not. The term adenocarcinoma may be used only if it refers strictly to the histological pattern.

The term bronchogenic carcinoma, most commonly used for tumours of the lower respiratory tract, is inadequate because there are many true tumours of pulmonary parenchyma, in addition to bronchogenic tumours. If one wishes to use only one comprehensive term for all tumours of the lower respiratory tract, the best term would then be broncho-pulmonary cancer (or carcinoma or tumour).

These considerations are important, not only for the classification of pulmonary tumours but also for the problems which must be faced in the research on occupational and atmospheric lung carcinogens.

### SUMMARY

Four hundred and fourteen cases of lung tumours studied on surgical resections have been reviewed. They have been subdivided into hilar-parahillar and peripheral tumours according to whether or not they were in obvious connection with bronchi greater than 2 mm. in diameter. Microscopically the tumours have been classified under four histological headings.

It was found that most small and squamous cell carcinomas were in connection with the bronchi and that most large cell carcinomas and adenocarcinomas were peripheral. Some histological aspects of adenocarcinomas strongly suggest that these tumours develop from bronchiolo-alveolar epithelium. Furthermore, anaplastic large cell carcinomas, including the giant cell ones, are histogenetically related to the adenocarcinomas.

The conclusion is reached that anaplastic large cell carcinomas and adenocarcinomas are tumours developing from bronchiolo-alveolar epithelium.

I am much indebted to Professor G. Mottura for his interest and encouragement in the preparation of this paper and to Professor E. H. Cooper and Mrs. G. M. Bonser for critical advice and their help in preparing the manuscript.

### REFERENCES

Amprino R.—(1937) *Archo ital. Anat. Embriol.*, **38**, 447.
Belanger, R., La Fleche, L. R. and Picard, J. L.—(1964) *Thorax*, **19**, 1.
Brooks, R. E.—(1966) *Am. Rev. resp. Dis.*, **94**, 112.
Bucher U. and Reid, L.—(1961*a*) *Thorax*, **16**, 207.—(1961*b*) *Thorax*, **16**, 219.
Campobasso, O.—(1963) *Riv. Anat. patol. Oncol.*, **24**, 476.
Campobasso, O. and Ferrara, L.—(1963) *Cancro*, **16**, 706.
Collet, A.—(1965) *Archo ital. Anat. Istol. patol.*, **39**, 119.
Delarue, J., Abelanet, R. and Paillas, J.—(1964) *Bronches*, **14**, 492.

DUBREUIL, G., LACOSTE, A. AND RAYMOND, R.—(1936) *Bull. Histol. appl. Physiol. Path.* **13**, 235.
DUNNILL, M. S.—(1962) *Thorax*, **17**, 329.
GARLAND, L. H., BEIER, R. L., COULSON, W., HEALD, J. H. AND STEIN, R. L.—(1962) *Radiology*, **18**, 1.
GUILLAN, R. A. AND ZELMAN, S.—(1966) *Am. J. clin. Path.*, **46**, 427.
FISCHER, W.—(1949) *Zentbl. allg. Path.*, **85**, 193.
FRIEDBERG, E. C.—(1965) *Cancer, N.Y.*, **18**, 259.
HELLSTROM R. H. AND FISHER, E. R.—(1963) *Cancer, N.Y.*, **16**, 1080.
HERMAN, D. L., BULLOCK, W. K. AND WAKEN, J. K.—(1966) *Cancer, N.Y.*, **19**, 1337.
KOTIN, P. AND WISELEY, D. V.—(1963) *Prog. exp. Tumor Res.*, **3**, 186.
KRAHL, V. E.—(1963) *Archs envir. Hlth*, **6**, 37.
KREYBERG, L.—(1962) *Acta path. microbiol. scand.*, Suppl. 157.
LEESON, T. S. AND LEESON, C. R.—(1964) *J. Anat.*, **98**, 183.
LISA, J. R., TRINIDAD, S. AND ROSENBLATT, M. B.—(1965) *Am. J. clin. Path.*, **44**, 375.
LOOSLI, C. G. AND POTTER, E. L.—(1951) *Anat. Rec.*, **109**, 320.
MCDONALD, J. R., MCBURNEY, R. P., CARLISLE, J. C. AND PATTON, M. M.—(1951) *J. thorac. Surg.*, **22**, 62.
MELNICK, P. J., ROSEN, S. H., HOWELL, C. W. AND HEISER, P.—(1964) *Am. J. Path.*, **44**, 39a.
MOLLO, F., CAMPOBASSO, O. AND CANESE, M. G.—(1967) *Atti X Congr. Soc. ital. patol.*, parte II, IDOS, Milano (in press).
MOTTURA, G. AND CAMPOBASSO, O.—(1966) *Minerva pneumol.*, **5**, 40.
NASH, A. D. AND STOUT, A. P.—(1958) *Cancer, N.Y.*, **11**, 369.
POLICARD, A., COLLET, A. AND PERGERMAIN, S.—(1957) *Path. Biol., Paris*, **33**, 385.
RAEBURN, G. AND SPENCER, H.—(1953) *Thorax*, **8**, 1.
REID, J. D. AND CARR, A. H.—(1961) *Cancer, N.Y.*, **14**, 673.
ROSEMOND, G. P., BOUCOT, K. R. AND AEGERTER, E.—(1951) *J. thorac. Surg.*, **22**, 99.
SCHULZ, H.—(1959) 'Die submikroskopische Anatomie und Pathologie der Lunge'. Berlin-Gottingen-Heidelberg (Springer-Verlag).—(1963) *Lab. Invest.*, **12**, 616.
SPENCER, H. AND SHORTER, R. G.—(1962) *Nature, Lond.*, **194**, 880.
STEWART, H. L.—(1959) in F. Homburger 'The physiopathology of cancer', 2nd edition, New York (Hoeber-Harper).
WADDELL, W. R.—(1949) *Archs Path.*, **47**, 227.
WALTER, J. B. AND PRYCE, D. M.—(1955) *Thorax*, **10**, 107.
WHITWELL, F.—(1961) *Br. J. Cancer*, **15**, 429.
WILLSON, H. G.—(1928) *Am. J. Anat.*, **41**, 97.

A full discussion of many aspects of lung development is contained in 'Development of the Lung' (1967) edited by A. V. S. de Reuck & R. Porter. London (A. & J. Churchill).


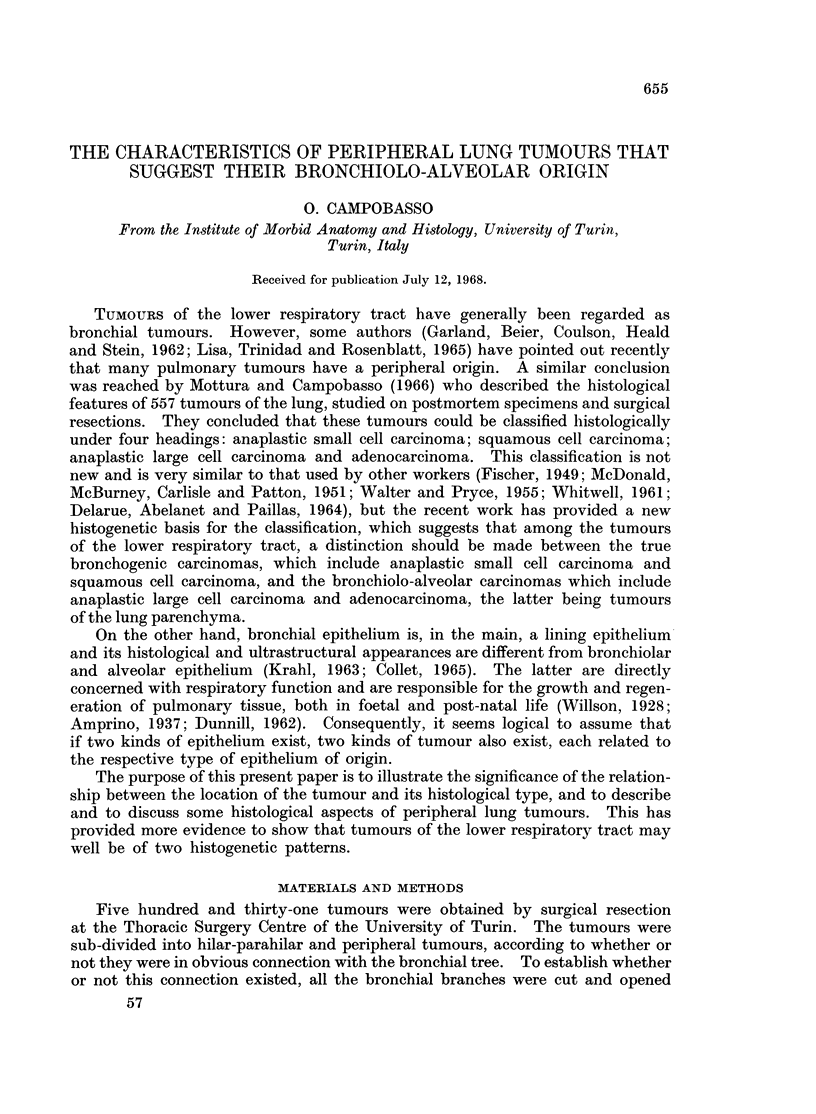

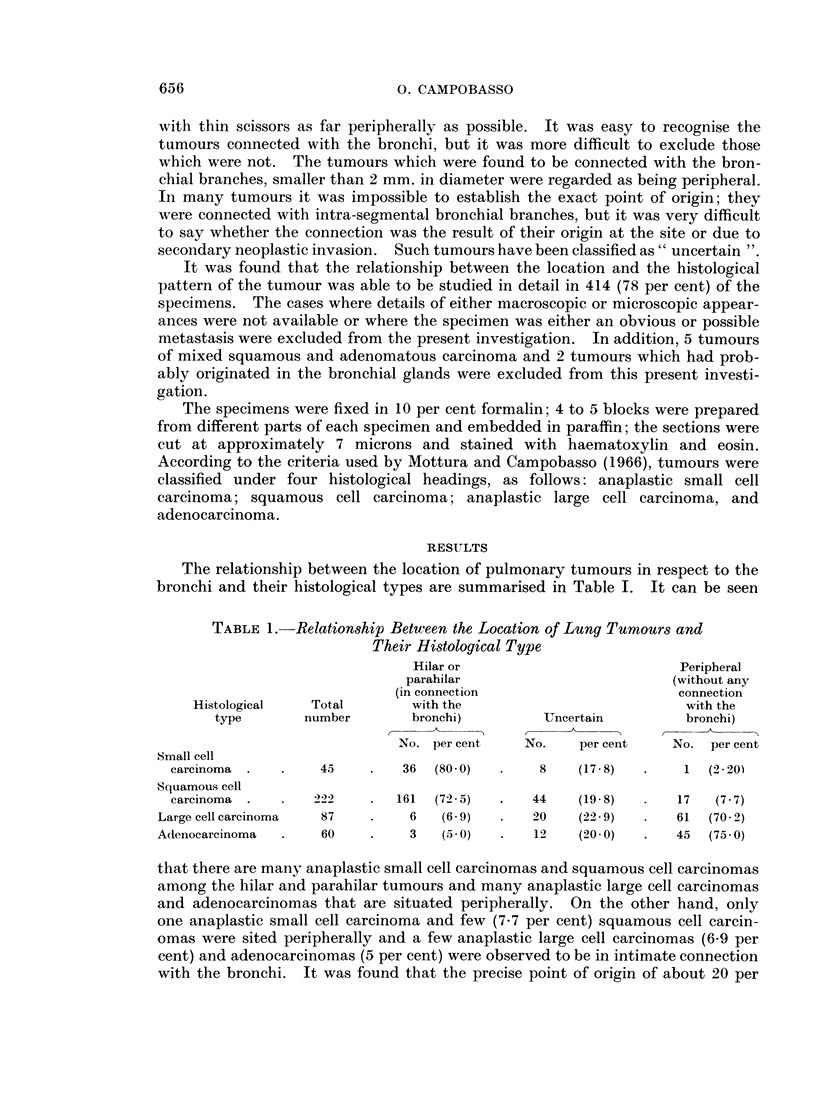

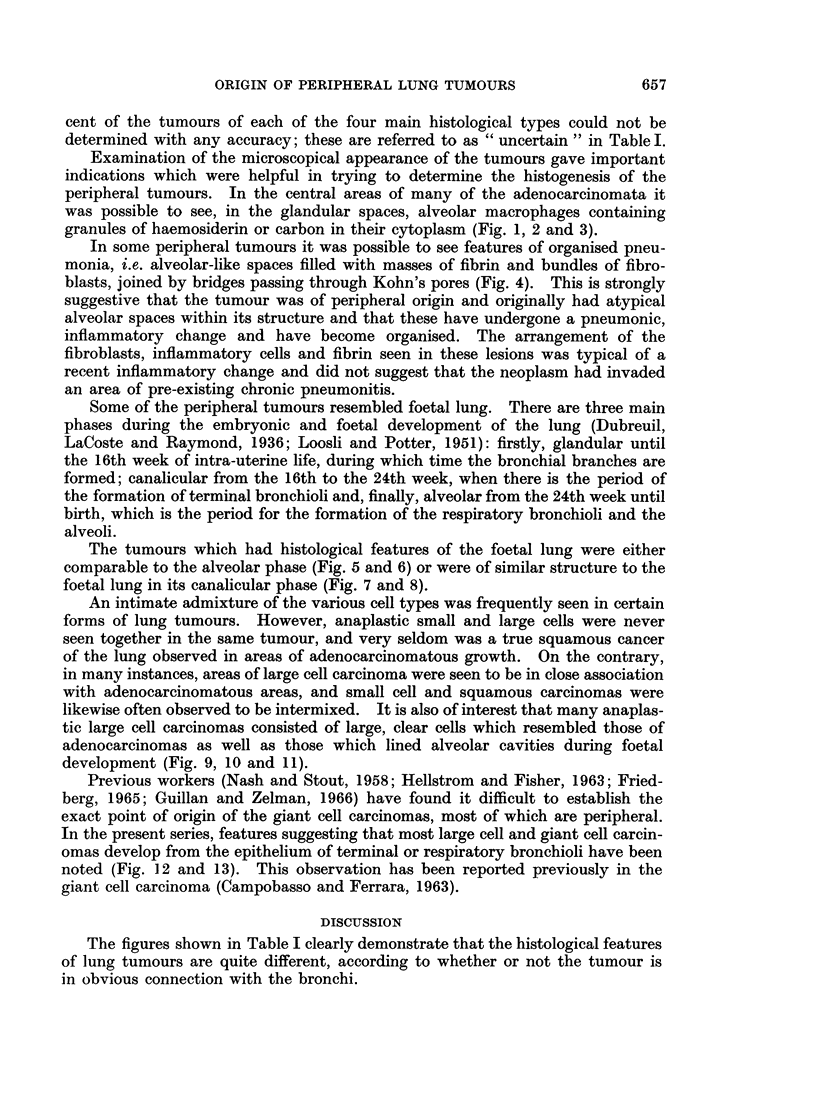

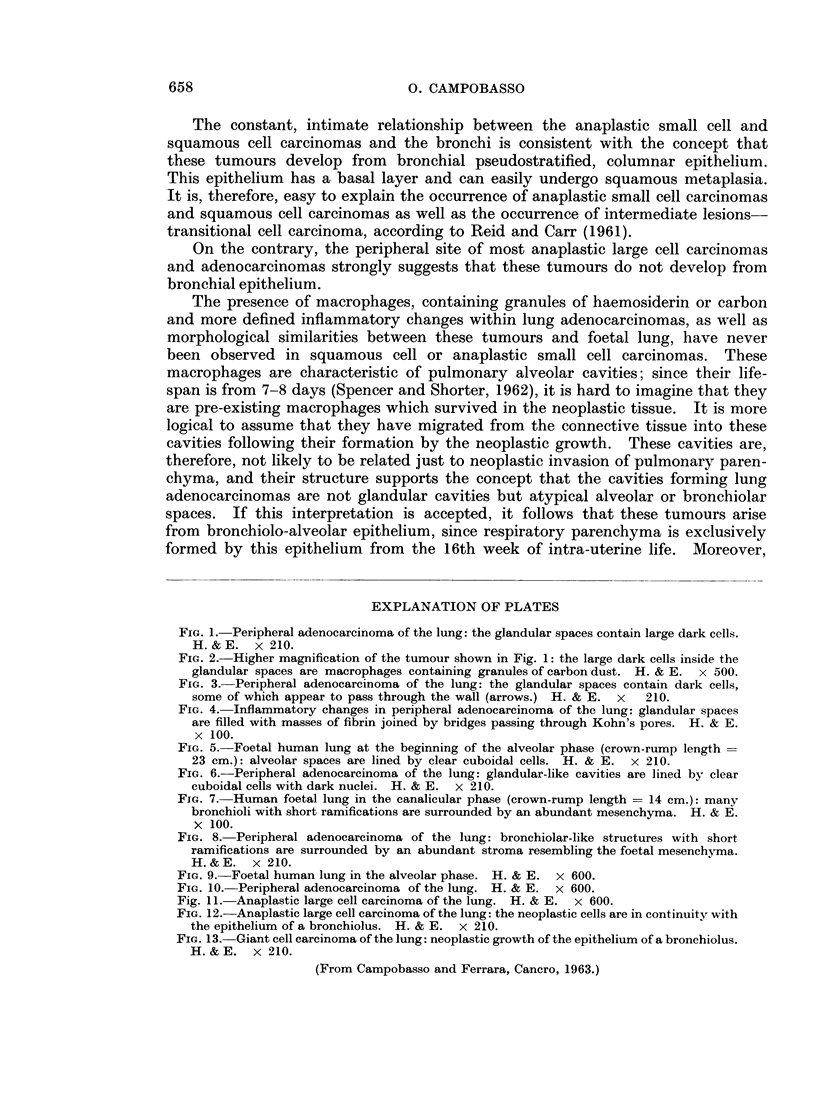

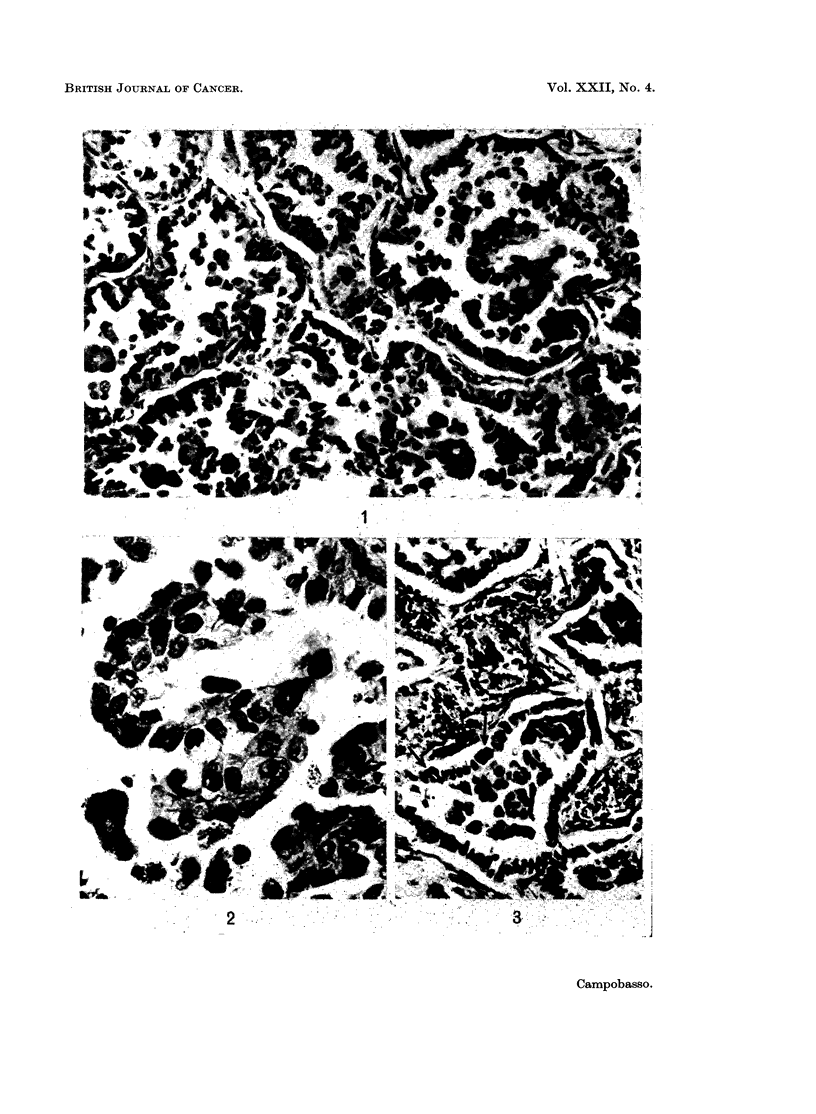

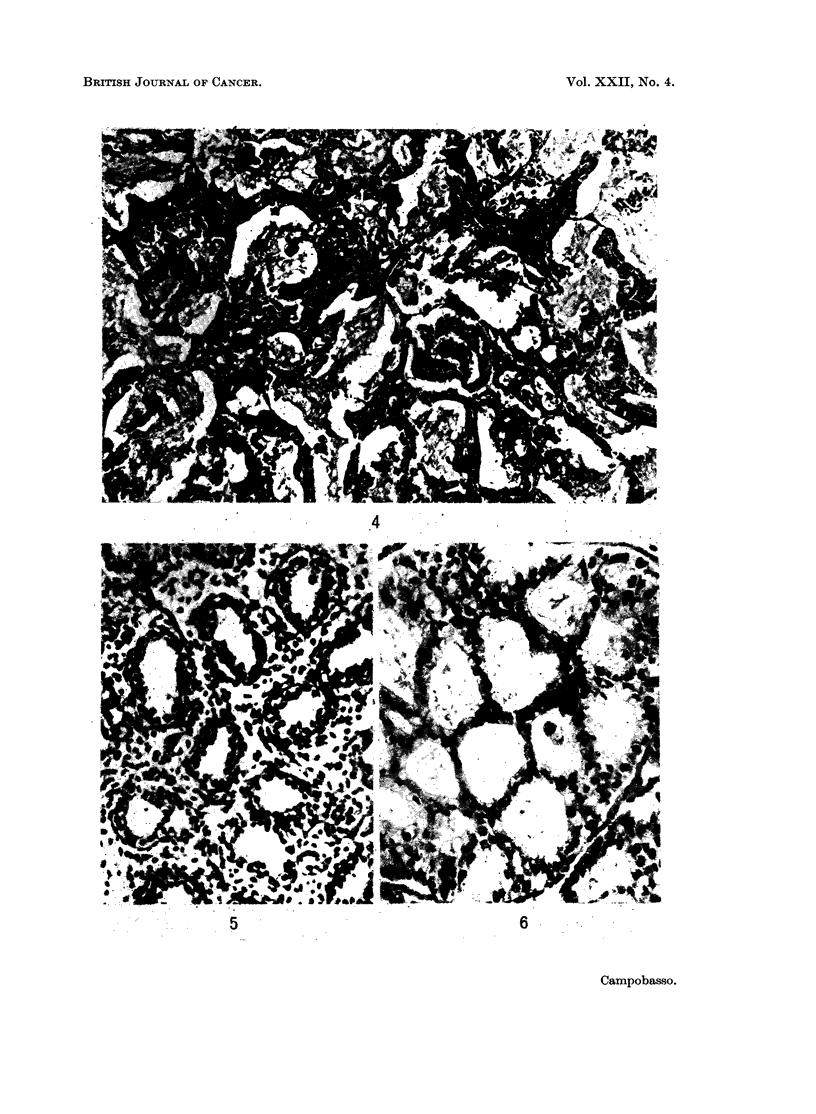

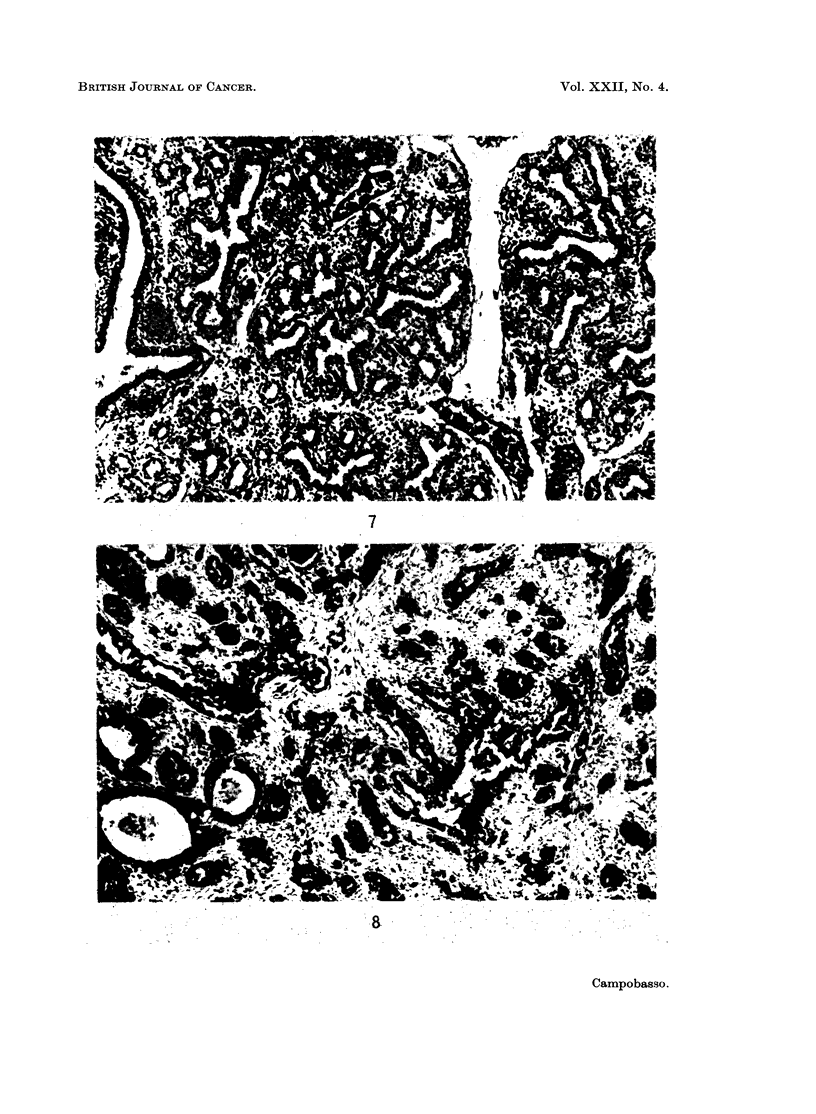

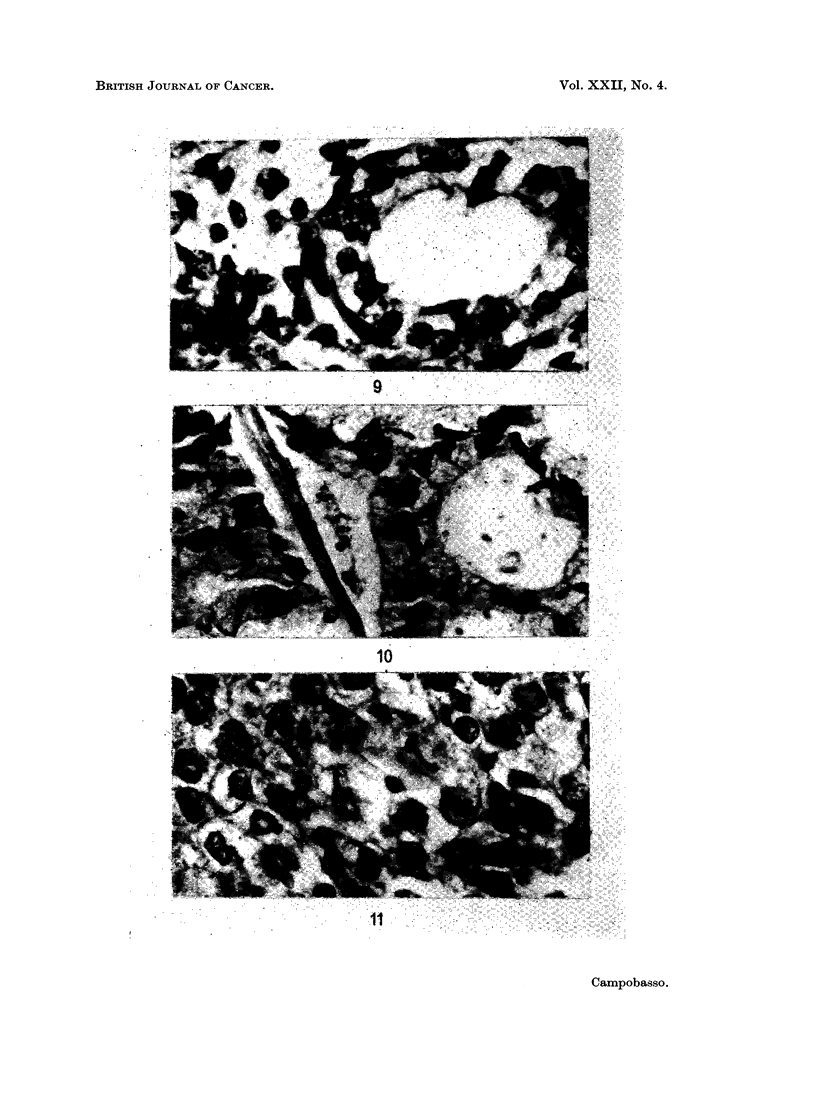

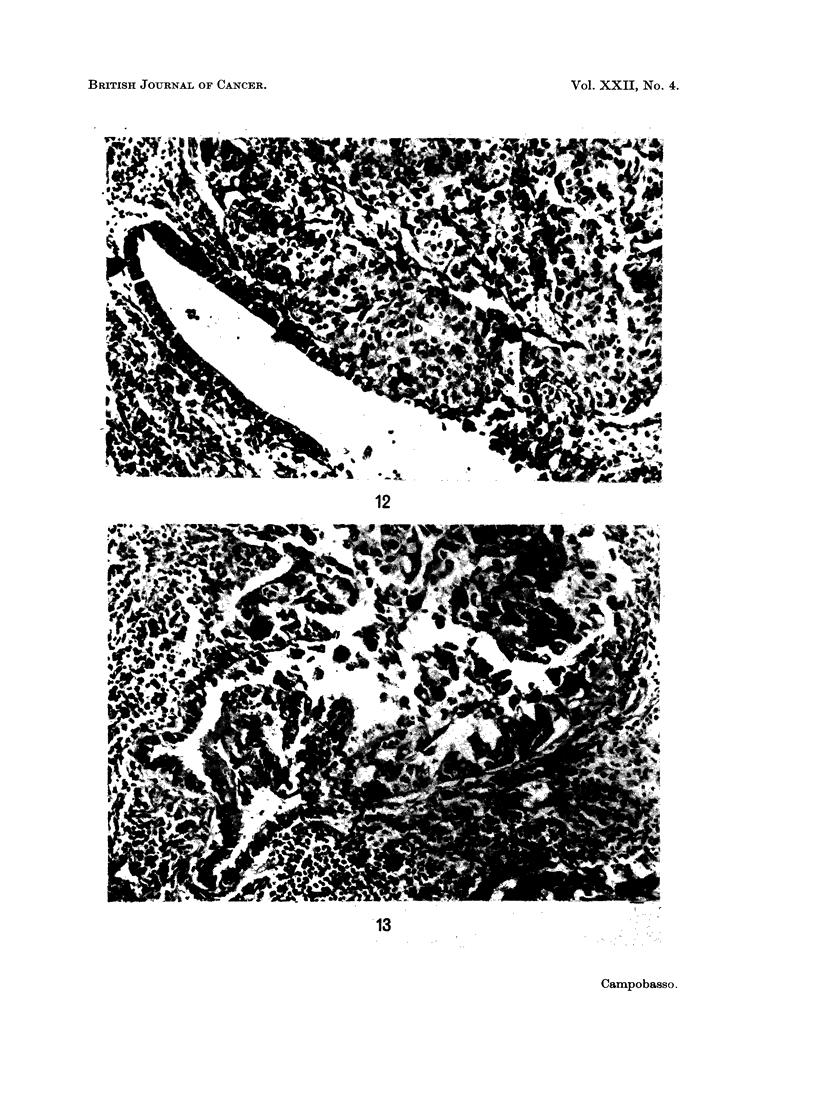

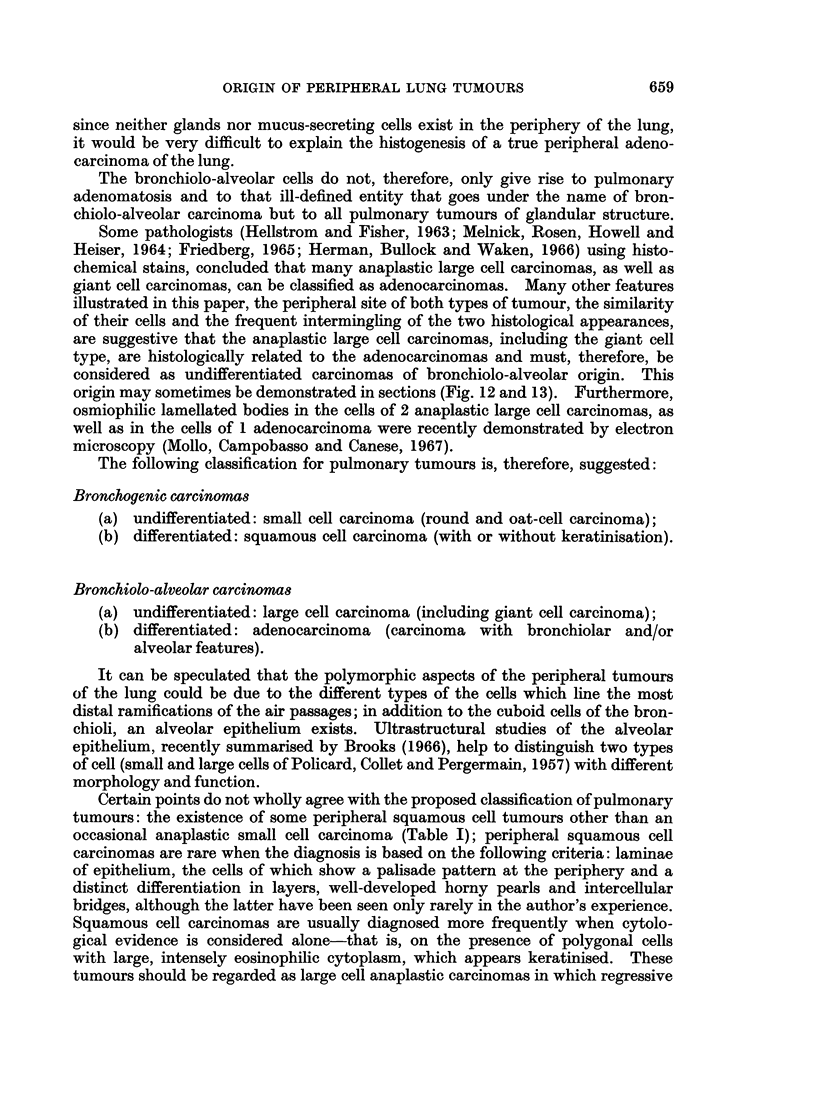

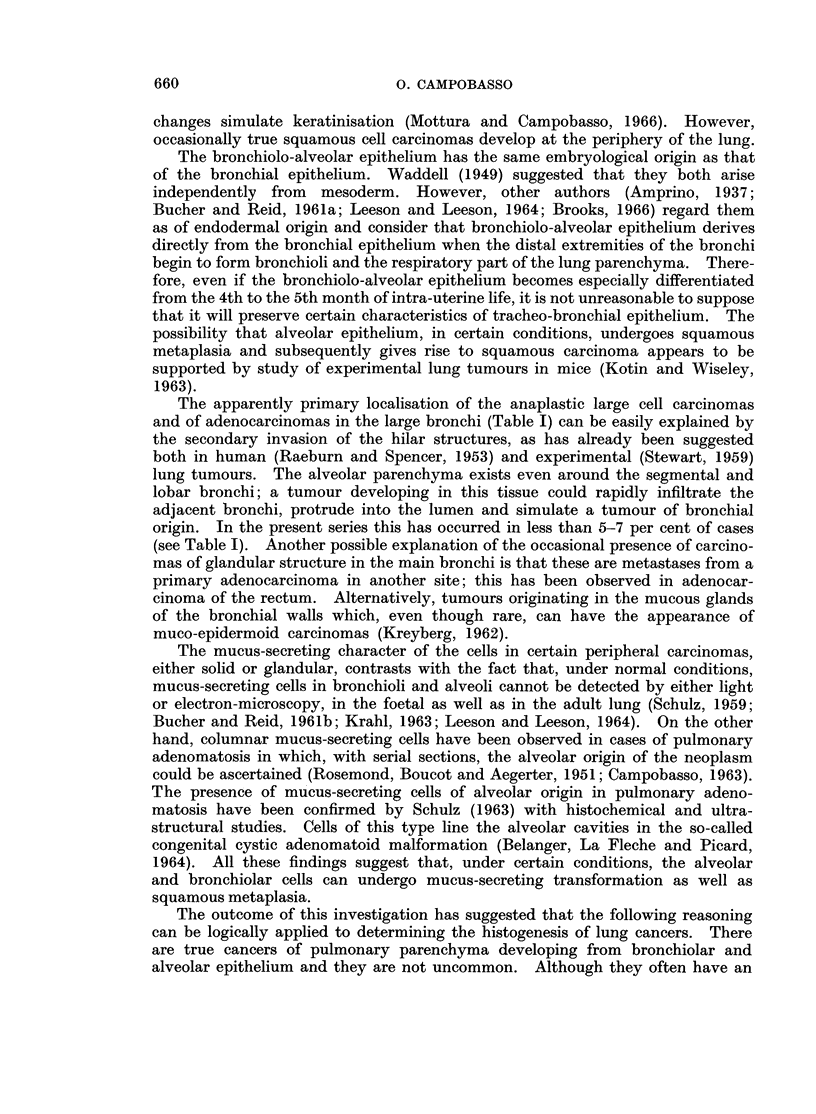

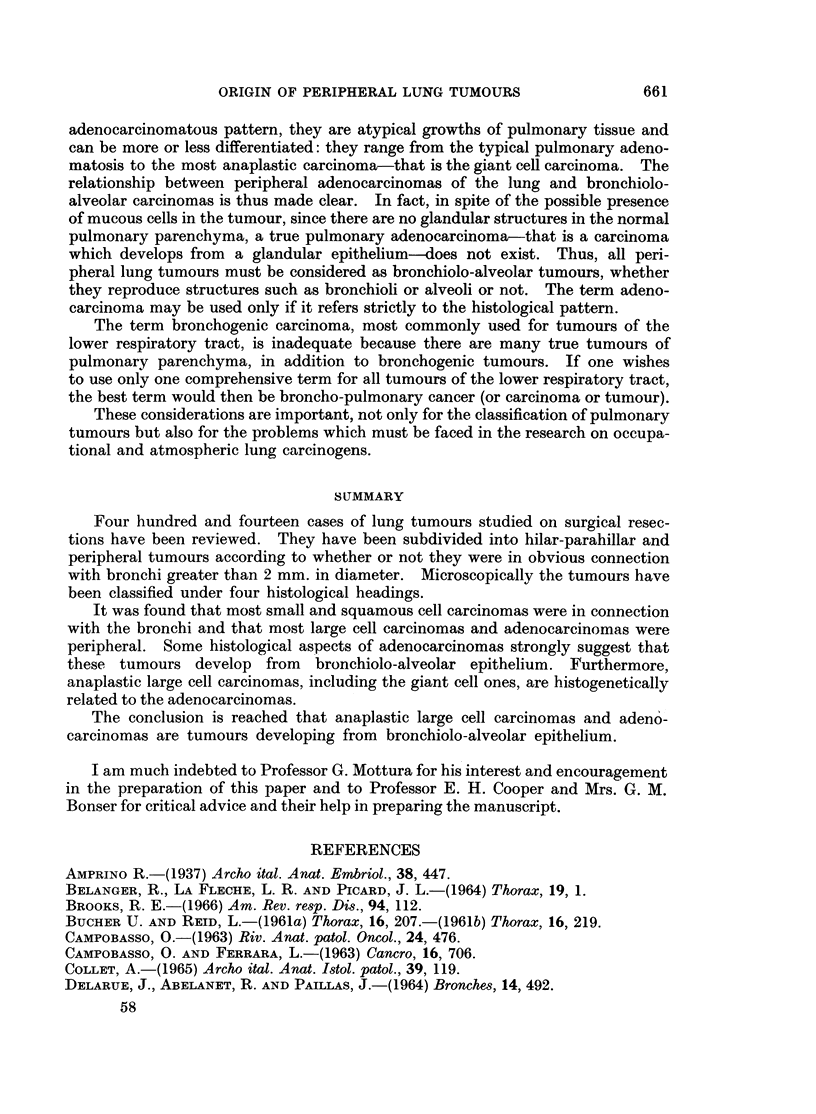

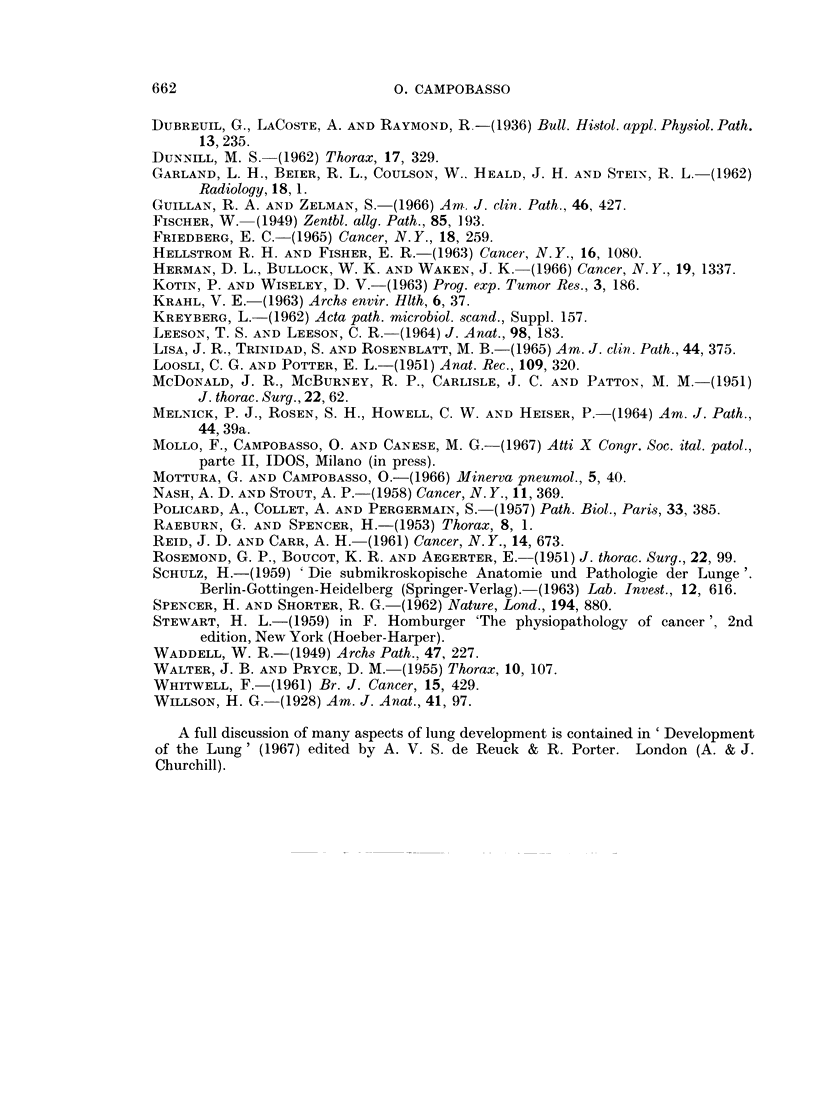


## References

[OCR_00495] BUCHER U., REID L. (1961). Development of the mucus-secreting elements in human lung.. Thorax.

[OCR_00498] CAMPOBASSO O., FERRARA L. (1963). IL CARCINOMA A CELLULE GIGANTI DEL POLMONE (PRESENTAZIONE DI TRE CASI. Cancro.

[OCR_00520] FRIEDBERG E. C. (1965). GIANT-CELL CARCINOMA OF THE LUNG: A DEDIFFERENTIATED ADENOCARCINOMA.. Cancer.

[OCR_00513] GARLAND L. H., BEIER R. L., COULSON W., HEALD J. H., STEIN R. L. (1962). The apparent sites of origin of carcinomas of the lung.. Radiology.

[OCR_00517] Guillan R. A., Zelman S. (1966). Giant-cell carcinoma of the lungs. An analysis of 12 cases.. Am J Clin Pathol.

[OCR_00522] HELLSTROM H. R., FISHER E. R. (1963). GIANT CELL CARCINOMA OF LUNG.. Cancer.

[OCR_00524] Herman D. L., Bullock W. K., Waken J. K. (1966). Giant cell adenocarcinoma of the lung.. Cancer.

[OCR_00525] KOTIN P., WISELEY D. V. (1963). PRODUCTION OF LUNG CANCER IN MICE BY INHALATION EXPOSURE TO INFLUENZA VIRUS AND AEROSOLS OF HYDROCARBONS.. Prog Exp Tumor Res.

[OCR_00529] LEESON T. S., LEESON C. R. (1964). A LIGHT AND ELECTRON MICROSCOPE STUDY OF DEVELOPING RESPIRATORY TISSUE IN THE RAT.. J Anat.

[OCR_00531] Lisa J. R., Trinidad S., Rosenblatt M. B. (1965). Site of origin, histogenesis, and cytostructure of bronchogenic carcinoma.. Am J Clin Pathol.

[OCR_00534] McDONALD J. R., McBURNEY R. P., CARLISLE J. C., PATTON M. M. (1951). The significance of cell types in bronchogenic carcinoma.. J Thorac Surg.

[OCR_00547] NASH A. D., STOUT A. P. (1958). Giant cell carcinoma of the lung; report of 5 cases.. Cancer.

[OCR_00554] ROESMOND G. P., BOUCOT K. R., AEGERTER E. (1951). Solitary pulmonary adenoma (focal pulmonary adenomatosis): a three-year follow-up after resection.. J Thorac Surg.

[OCR_00559] SPENCER H., SHORTER R. G. (1962). Cell turnover in pulmonary tissues.. Nature.

[OCR_00567] WALTER J. B., PRYCE D. M. (1955). The histology of lung cancer.. Thorax.

[OCR_00568] WHITWELL F. (1961). The histopathology of lung cancer in Liverpool: a survey of bronchial biopsy histology.. Br J Cancer.

